# The Great Escape: mRNA Export through the Nuclear Pore Complex

**DOI:** 10.3390/ijms222111767

**Published:** 2021-10-29

**Authors:** Paola De Magistris

**Affiliations:** Bionanoscience Department, Kavli Institute for Nanoscience, Delft University of Technology, 2629HZ Delft, The Netherlands

**Keywords:** mRNA, mRNP, NPC, nuclear transport, nuclear export, Mex67/MTR2, NXF1/NXT1, Sub2/UAP56, Yra1/Aly

## Abstract

Nuclear export of messenger RNA (mRNA) through the nuclear pore complex (NPC) is an indispensable step to ensure protein translation in the cytoplasm of eukaryotic cells. mRNA is not translocated on its own, but it forms ribonuclear particles (mRNPs) in association with proteins that are crucial for its metabolism, some of which; like Mex67/MTR2-NXF1/NXT1; are key players for its translocation to the cytoplasm. In this review, I will summarize our current body of knowledge on the basic characteristics of mRNA export through the NPC. To be granted passage, the mRNP cargo needs to bind transport receptors, which facilitate the nuclear export. During NPC transport, mRNPs undergo compositional and conformational changes. The interactions between mRNP and the central channel of NPC are described; together with the multiple quality control steps that mRNPs undergo at the different rings of the NPC to ensure only proper export of mature transcripts to the cytoplasm. I conclude by mentioning new opportunities that arise from bottom up approaches for a mechanistic understanding of nuclear export.

## 1. Introduction

The passage of mRNAs to the cytoplasm occurs through the nuclear pore complexes (NPCs) that, with their ~125 MDa, make the cellular largest protein complex, spanning the double nuclear envelope. This review is aimed at summarizing our current knowledge of the basic mechanisms behind protein-coding mRNA export from the nucleus, encompassing the maturation, quality control and packing steps, before discussing its interaction and remodelling within the NPC.

In spite of its defined chemical composition, RNA is a versatile macromolecule. Cellular RNA can vastly differ in many aspects, e.g., by which polymerase it is produced (PolI, PolII or PolIII), which processing it undergoes subsequently, whether it is translated or not, like for messenger RNA versus non-coding RNA [1], its final cellular localization (nuclear, cytoplasmic, or mitochondrial), and its length, which can vary substantially from tens of nucleotides for miRNA [2], to snRNA [3], to rRNA [4], and to kilobases for mRNA. The subset of messenger RNAs (mRNAs) are the most well-known. Historically, this molecule has been spotlighted for its role as a mediator of the genetic information encoded in DNA to provide a template for translation of protein.

Importantly, mRNA molecules in cells are not found on their own, but form ribonucleoprotein complexes (mRNPs) by associating with RNA binding proteins (RBPs), proteins that are crucial for the mRNA metabolism. Recent years have seen major progress in the identification of RBPs, revealing that they correspond to a large fraction of the cell proteome [5]. Yet, elucidating in detail the molecular mechanisms underlying how mRNA production, export and regulation are linked to RBPs represents a very difficult task. This is due to the large variety of RBPs, to their RNA sequence specificity or lack thereof, to the variety of mRNAs, but also because of the extremely high number of possible combinatorial interactions between mRNAs and RBPs [6]. Crucial information that could help organize this huge set lies in the 3D organization of the mRNPs. Recent years have provided key new insights into mRNP morphology, but mostly in the context of translation [7,8].

**Figure 1:**
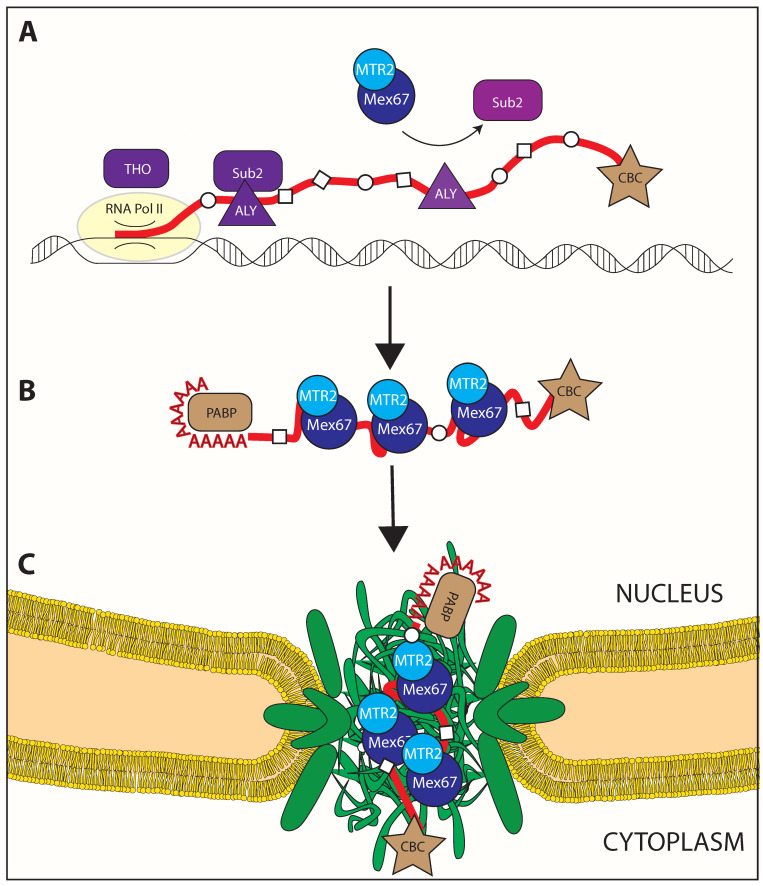
mRNA export is intertwined with its processing. Correct maturation of export competent mRNPs relies on sequential steps that go hand-in-hand with transcription initiation, capping, and processing. (**A**) In yeast, the mature mRNP is characterized by loading of Mex67/MTR2 (in blue), the FG-interacting transporter of the bulk nuclear RNA (in red), and presence of multiple RNA binding proteins (in white). Its loading to the RNA is not direct, and requires adaptors which are themselves loaded onto the transcribing RNA while it is produced. (**B**) Once loaded, the Mex67/MTR2 heterodimer mediates exit of the mRNP from the nucleoplasm via fast, transient interactions with the multiple FG repeats within the central channel of the NPC (in green) (**C**), eventually enabling the mRNP passage through the selective barrier into the cytoplasm.

As a consequence of mRNA processing, defined RBPs will be co-transcriptionally deposited onto the pre-mRNA at specific positions. The result is the formation of mRNPs with a specific set of RNPs binding to mRNAs that will influence the downstream events, assembled in a highly ordered process. The process leading to an export competent mRNP is defined maturation. Therefore, as mRNAs are being transcribed in the nucleus, it is processed and packaged into mRNPs, which change their structure as additional RNA is transcribed, as additional proteins associate, and as processing modifies the RNA. A defined set of RNPs are needed for every aspect of the mRNP control, including export, localization in the cytoplasm, quality control, stability, engagement with the translation machinery, and degradation. The loading of transport protein generates mature mRNPs, which are targeted to the nuclear pore complex and translocated (Figure 1).

This review intends to highlight recent findings related to mRNP maturation into export-competent complexes, the key interactions with members of the nuclear pore complex (recapitulated in Table 1), and the perspective of novel approaches relating to questions on the mRNA export process.

## 2. Nuclear Processing of RNA

### 2.1. Remodeling by RNA Binding Proteins (RBPs)

Each mRNA in the cell is not present in free form, but is bound by a specific set of multiple RNA binding proteins (RBPs) at a certain moment in its lifetime. This coverage has a double function: on the one side, it prevents RNA from premature degradation, avoiding attack by the cellular RNA surveillance systems [9]. Furthermore, it provides a regulation platform that allows the cell to monitor the maturation step and localization of the mRNA, finely controlling its translation state [10,11,12] and eventually its degradation [13]. While the mRNP proteome has been relatively well characterized, much less is known about its spatiotemporal organization.

Elucidation of the molecular mechanism of RNA structure, transport, and translation involves the challenging task of identification and characterization of the RBPs that are involved. Thousands of putative RBPs have been identified and deduced from sequence homologies. However, only a subset of them has been validated in vivo, in human [14,15] or mouse cell lines [16].

The RBPs bind to localization elements, also known as RNA transport signal (RTS), which are sequences of mRNA that are required for nuclear export that are located mainly in the 3′ untranslated region (UTR) [17,18]. Early work demonstrated the existence of such RTS elements in neuronal mRNAs, containing dendritic targeting sequences in the 3′ UTR region [14,19,20,21,22]. This binding strategy is not dissimilar from nuclear localization sequences (NLS) in proteins that require nuclear import [23]. Less frequently, the binding sites for RBPs can be also found in the 5′ UTR coding sequences, or in introns (reviewed in [24,25]). A comprehensive attempt at an atlas of RTSs has been tackled as part of the ENCyclopedia Of DNA Elements (ENCODE) project (https://www.encodeproject.org/, last access 1 March 2021), launched in 2003, currently in phase III [26]. Extending this project will greatly contribute to define to what extent the combinatorial code of RTSs and their target RBPs drives the final localization of mRNPs, and their global effect on cell physiology.

The spatial arrangement of RPBs along the RNA molecule has a central role in the overall regulation of the message translocation and translation. Some RBPs show sequence specificity, while others (like Mex67/MTR2) do not. It is estimated that mRNPs are composed by 50 to 80% proteins in weight, but accounting for a relatively lower RNA coverage (~50%) [27]. In recent years, mutations in genes that encode for RBPs have emerged as important factors in neurological diseases such as amyotrophic lateral sclerosis (ALS), pointing to a pivotal role of mRNA export and localization to human health [28]. These defects have been identified both as primary nucleotide sequences mutations, but also as defective secondary structures that fail to target RNPs for RNA binding [29,30,31].

Members of the spliceosome and the Exon Junction Complex (EJC) can be included among RBPs involved in mRNA maturation. The capping binding complex (CBC) includes some of the first RBPs recruited on the nascent RNAPolII transcript [32] (see Figure 1). It is composed by the heterodimer Cbc1/Cbc2 in yeast, homologue to metazoan Cbp20/Cbp80 and is required for successful mRNA export [33].

The spliceosome is the collective name given to a ribonucleoprotein complex responsible for splicing, the mRNA maturation step during which defined trancript sequences, introns, are removed in a controlled manner. A class of RBPs named SR proteins, rich in serine and arigine residues, has been first implicated in splicing [34], although they were more recently pinpointed as a link between completed mRNA splicing and export [35]. In humans, among the 12 known SR proteins, some are removed from the mRNP as soon as splicing is successfully completed, while others are exported to the cytoplasm together with it [36]. On the contrary, in yeast, all the three known SR proteins (Npl3, Gbp2 and Hrb1) remain attached to the mRNP all over the export process, and are only released in the cytoplasm [37,38,39], although it is not clear how they are released [40].

Following intron removal, the coding transcript sequences, the exons, must be joined to form the mature transcript. This link is achieved by the EJC, which is deposited upstream of the exon–exon junction and has been suggested to serve as a docking platform for mRNA export factors [41,42,43], compatibly with the finding that members of the EJC complex remain attached to mRNP in its journey through the NPC to the cytoplasm, where they are finally displaced from the transcript [44,45]. The prevalence of splicing in higher eukaryotes suggests a conserved mechanism for EJC based maturation: 90% of human genes contain introns, and although the same is true of only 6% of *S. cerevisiae* genes, 30% of the yeast transcriptome is composed by mRNAs that undergo splicing [46].

Indeed, in human, ALY/REF loading is achieved thanks to a mixed contribution of both the cap binding complex (CBC) and of the exon junction complex [43], and with the mediation of export factor Chtop, which cooperates with Aly/REF within the TREX complex [43,47,48]. The centrality of the EJC in coordinating the deposition of export factors on the mRNA [43,48,49,50,51] led to revisit previous models according to which attachment of the polyA tail at the 3′ end, and the 3′ poly-A binding proteins, was thought to be responsible for signalling full maturation of the transcript.

We are still far from a complete overview of how mRNA and proteins organize into an mRNP, and even less is known about how its protein composition changes during its journey. The finding that the compaction state of RNA in the cytosol is depending on ribosome binding in the cytosol [7,8], opened a new wave of interest towards the general principles underlying the 3D arrangement of mRNPs during their lifetime.

### 2.2. Mex67/MTR2 Is the Key Driver of mRNA Export

Despite the heterogeneity of mRNAs in terms of size, maturation state, associated RNPs, splicing, and copy number, export of the majority of mRNAs is thought to be mediated by a non-karyopherin export receptor, the heterodimeric Mex67/Mtr2 in budding yeast (or NXF1/NXT1 or TAP/p15 in humans) (Figure 1). In addition to Mex67/MTR2, a small subset of transcripts is known to be exported by the karyopherin-like protein CRM1, which is mainly responsible for export of Nuclear Export Signal (NES)-containing proteins [52]. CRM1 is not an RNA binding protein, and its binding to RNA happens to yet unidentified adaptors [53]. The bulk of polyadenylated mRNA export is carried out by Mex67/MTR2, which will be the focus of this review.

The fact that this heterodimeric protein complex is required for mRNA export was first based on observations that (i) Mex67/MTR2 deletion in yeast is lethal, while a *mex67* thermosensitive mutant promotes accumulation of polyadenylated RNA in the yeast nucleus [54,55], (ii) that its human homologue, NXF1/NXT1 can rescue the yeast lethal deletion50, and (iii) that NXF1/NXT1 mediates nuclear export of CTE (constitutive transport element)-containing RNA, a short retroviral sequence that is able to bypass host export [56,57].

**Figure 2:**
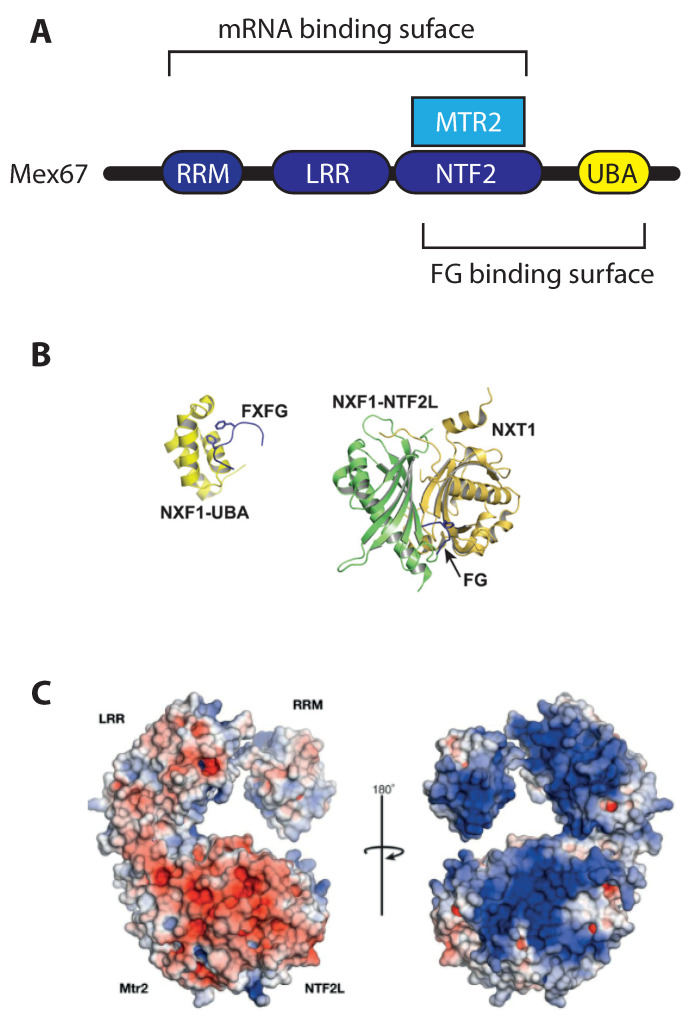
Mex67/MTR2. Mex67/MTR2 (domain schemed in (**A**)) mediates the bulk RNA export in *S. cerevisiae*. It interacts with FG nucleoporins via its UBA domain (**B**) and with RNA, with its RRM, LLR and NTF2 domains, that form an extended, positively charged binding surface (**C**). Panel (**B**) from [59], and panel (**C**) from [58].

In order for Mex67/MTR2 to function as an mRNP export receptor, it must bind both RNA cargo as well as the FG-repeat in nucleoporins. Structural arrangement of Mex67/MTR2 is depicted in Figure 2. Yeast Mex67 and human NXF1 share a similar domain organization (Figure 2A) that includes, from N- to C-terminal: an RNA recognition motif (RRM), a leucine-rich region (LRR), an NTF2-like domain (NTF2L) and an ubiquitin-associated (UBA) domain. Mtr2 remains associated with Mex67 via its own NTF2-like fold, which binds to the NTF2L domain of Mex67. As for cargo binding, RRM, LRR, and NTF2L domains seem to be able to participate [58]. A specificity for ribonucleic sequences has never emerged, which is consistent with the expectations for the export receptor of the whole transcriptome. Due to the lack of sequence specificity, the loading of Mex67/MTR2-NXF1/NXT1 to the bulk mRNA is mediated by adaptors, which generate conformational changes in the secondary structure of the mRNA [29,30,31]. Due to the low specificity of Mex67MTR2 binding to RNA, and the complexity of the protein composition of the mRNPs, which vary during maturation, the exact arrangement of this binding and copy number of export receptor(s) per mRNP during the maturation process remain unsolved.

As for FG-repeat recognition, which enables Mex67/MTR2 to export the mRNP through the NPC, Mex67/NXF1 is capable to interact with to a plethora of FG motives from different nucleoporins [60,61,62,63] via its NTF2L [64] and UBA domains [65] (Figure 2B). The binding of Mex67/MTR2 to either type of FxFG or GLFG repeats raises the question of whether any specificity—or preference for repeat type exist: per se, the UBA domain of Tap shows higher affinity for FxFG cores compared with GLFG cores [66], and in vivo, the transporter is sensitive to deletions of different nucleoporins with respect to their relative position into the NPC [67]. Binding between the isolated UBA domains of Mex67/NXF1 and FG-repeats quantifies around a K_D_ of tens of μM [66], identifying its binding to FG-nups as relatively weaker than the karyopherins in similar biochemical assays [68]. The exchange of FG-repeats from the export receptor in the hundreds of milliseconds range can then be easily explained [69]. The low-specificity of binding entails that the mode and the stoichiometry of loading of Mex67/MTR2 on RNA has remain structurally elusive, without a clear understanding of the transport function of Mex67/MTR2.

The structural studies on Mex67/MTR2 are likely to shed some light on the interactions that occur with the mRNA in the mRNP on one side, and with FG-nups during transport on the other. For example, it has been found that two heterodimers of NXF1/NXT1 can also form a tetrameric assembly [58]: this conformation provides a double-surface arrangement, with a positively charged platform for RNA binding on one side, while providing its hydrophobic UBA domains freedom of access for FG-domain binding on the opposite face of the heterotetramer, which is supported by surface charge analysis in Mex67/MTR2 [70]. Physical proximity of more than one FG binding site could provide another means to further facilitate the export; yet, notably, the same study reports that mutations that interfere with the formation of the heterotetramer surface reduce both CTE-RNA and pre-60S ribosomal subunit export in vivo, but do not result in nuclear accumulation of poly(A) RNA. Possibly, if the domain swapping facilitates endogenous mRNA export, is not a strict requirement; nevertheless, the ribosomal RNA export defects suggest that the high-order Mex67MTR2 surface organization may rather be required for export of a set of non-messenger RNAs, such as the 5S rRNA in *S. cerevisiae*, or the hairpin-containing RNA in humans.

These recent findings of high-order structure–function relationship of mRNPs raise the intriguing possibility that the positioning and the stoichiometry of Mex67/MTR2 assembly on mRNA, possibly together with additional unidentified factors, may contribute to the success of nuclear export in unappreciated ways. Yet, the technical challenges posed by the complex structural arrangement and heterogeneity of the transcriptome has kept the precise function and the prevalence of the higher-order form of Mex67/MTR2 unclear in either yeast and human cells.

## 3. mRNP Export through the Nuclear Pore Complex

When the export competent mRNPs, loaded with Mex67MTR2-Tap/p15, successfully dock at the NPCs, they are translocated through the central channel via interaction with the FG domains within it, and released into the cytoplasm. The spatial arrangement of the FG repeats within the central channel is crucial for successful transport, and is dependent on the correct attachment of the central nucleoporins to the NPC core scaffold, embedded in the double nuclear envelope.

The approximately 120 megaDalton-sized macromolecular protein complex spans the double nuclear envelope (NE), forming an aqueous channel within the envelope. The nuclear pore complex (NPC) is therefore the gatekeeper of the nucleus, the mean by which mRNA, proteins, non-coding RNA, rRNA, and protein/complexes are shuttled in a controlled manner between in and outside of the nucleus. In a fully formed, functional interphasic nucleus, it is present in a few hundred to a few thousand copies, depending on the organism, cell type and on the cell cycle stage [71,72,73]. Each NPC is composed by about 450 copies of about ~30 different nuclear pore proteins called nucleoporins; these arrange together to form (a) a stiff scaffold encompassing the NE, which provides stability to the overall structure and serves as a mean to anchor (b) nucleoporins composing a structurally undefined central channel, enriched in phenylalanine-glycine motifs (FG-nups).

Specifically, the central channel nucleoporins are Nup98/Nup145, Nup116, Nup62/NSP1, Nup54/Nup57, and Nup45/Nup49. These central proteins form a mesh that collectively poses a limit to free diffusion of large macromolecules, but allows the translocation of others, specifically. This latter active transport requires the mediation of transport receptors that interacts with the FG-Nup mesh and mediates the translocation of even very large complexes, like ribosomal subunits that leave the nucleus. Apart from the receptors, in some cases, even regions of the protein cargo molecule proximal to the receptor binding site have been suggested to also contribute to efficient translocation [74].

Two more structures, the nuclear basket and the cytoplasmic filaments, must associate to the NE-embedded three ring core to complete the nuclear pore complex. Their proper attachment is required for the full functionality of the pore, and in our specific case, for proper docking and export of mRNA. The cytoplasmic filaments protrude from the NPC to the cytoplasm and are composed by nucleoporins Nup358, Nup214/Nup159 and Nup82. On the contrary, Nup2/Nup50, Nup153/NupNup60, Tpr/Mlp1, Nup98/Nup145, exclusively found on the nucleoplasmic side, are the members of the nuclear basket. Nevertheless, the historical use of terminology “basket” may be inaccurate: there is no evidence that the contacts between the nuclear basket proteins indeed create the shape of a basket. In fact, the nuclear basket components, in particular Nup50 and Tpr, have been proven to have the lowest residential time on NPC among nucleoporins [75,76]. In all likelihood, this feature is due to necessities of nuclear import/export shuttling, which requires the players of the cycle to be constantly recycled and available.

### 3.1. Remodeling upon Entering the Nuclear Pore Complex

Nucleocytoplasmic RNA export can be divided into (1) assembly of an export-competent mRNP, completed by loading of Mex67/MTR2, (2) remodelling at the nuclear basket, (3) trafficking through the NPC, and (4) release into the cytoplasm, where the mRNP is targeted for translation. mRNA biogenesis, maturation and packaging into export-competent mRNP complexes is subject to multiple quality control steps, which, importantly, are spatially separated.

### 3.2. Loading of Mex67/MTR2 on mRNP

**Figure 3:**
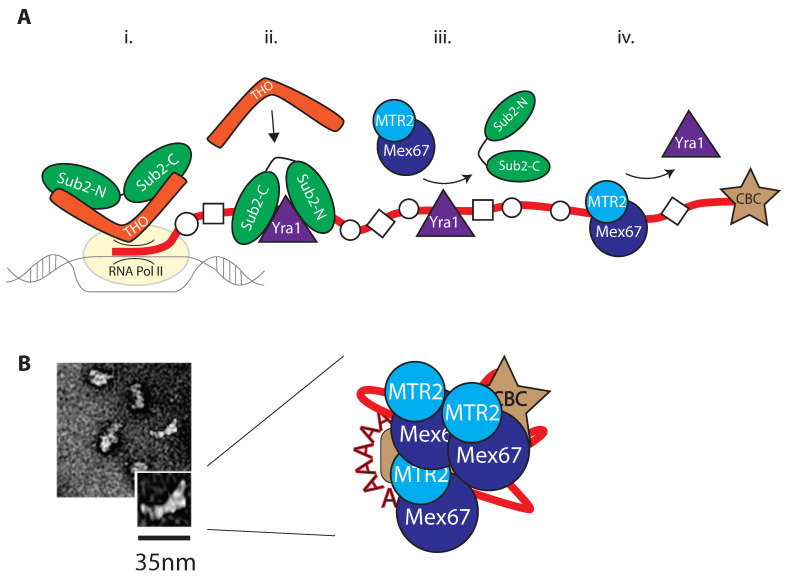
Preparing the mRNP for export. Mex67/MTR2 loading onto mRNA is the result of a concerted maturation process (schematized in (**A**)). (i.) THO, member of the TREX complex, anchors Sub2 in semi-open conformation to the nascent mRNP. (ii.) THO supports Sub2 anchoring to the mRNP, which clamps up in a closed conformation. (iii.) Mex67/MTR2 is recruited by Yra1, and its loading on mRNP displaces Sub2. (iv.) Yra1 leaves the mature mRNP following its ubiquitination. The process produces mature mRNP that vary from 15–35 nm in length (**B**). Panel (**B**) adapted from [78].

Loading of Mex67/NXF1 onto mRNP is the key step for production of mature mRNPs, and is achieved by members of the TRanscription EXport complex (TREX) (Figure 3). TREX is composed by yeast Sub2, THO, and Yra1, called UAP56, THO, and ALY, respectively, in humans (see also Table 1). Sub2/UAP56 is the RNA-dependent ATPase central to the remodelling process, which also has an ATPase dependant helicase activity; THO consists of a pentameric complex including proteins Hpr1, Mft1, Tex1, Tho2, and Thp2 (not shown in figure); and Yra1/ALY is an adaptor protein for Mex67/MTR2. The Sub2/UAP56 ATPase is a DEAD-box RNA helicase, so-called as it contains an Asp-Glu-Ala-Asp (DEAD) motif; it promotes key structural rearrangements of RNA as well as assembly/disassembly of RBP-RNA complexes, for which ATP hydrolysis is required. The structure of Sub2 has recently been solved in complex with members of the THO subcomplex, revealing two RecA-like domains (on the N-terminal and C-terminal moieties) [77].

TREX-dependent nuclear mRNP maturation occurs in steps, with loading of THO followed by Sub2 and Yra1, respectively (Figure 3A). TREX is already recruited at the transcription initiation level by associating to the phosphorylated RNA Pol II CTD [79] and it interacts with the Capping Binding complex (CBC) in human cells, and thereby facilitating recruitment of Sub2 to the transcription machinery by providing a 25 nm multivalent, composite scaffold [77,80]. The contact of THO with Sub2 occurs on both Sub2 RecA domains, inducing a “half-open” conformation [81].

The role of Sub2 is ultimately to promote loading of Yra1 onto the mRNP (Figure 3). Furthermore, a C-terminal fragment of Yra1 (Yra1-C) facilitates its own loading by stimulating Sub2 ATPase activity in vitro [82]. Aly has been first implicated in mRNA remodelling at the splicing level [82,83], therefore at a previous step of mRNP maturation. Recruitment of Yra1 precedes recruitment of the export receptor Mex67/Mtr2. Sub2, when found in a “closed” conformation, recognizes the sugar-phosphate backbone of RNA, indicating a sequence non-specific binding. It is to note that, just as it is the case for Mex67MTR2, the low sequence specificity for RNA matches the expectations of its role of bulk mRNA loader. Additionally, indeed, Sub2 and Yra1 cooperatively bind RNA in vitro [81] (Figure 3A). Sub2 likely dissociates from the mRNP at this step: as mentioned, Yra1 and Sub2 can both bind to RNA cooperatively, but the binding domains for Sub2 and Mex67/Mtr2 on Yra1 overlap [81,84], suggesting that Mex67/MTR2 loading promotes Sub2 removal. Nevertheless, Yra1, much like Sub2, is not a component of export-competent mRNP; it dissociates as a consequence of targeted ubiquitination [85,86] (Figure 3B). Further mechanistic details of how TREX executes loading of Mex67MTR2 have implicated the SR protein Gbp2 (see “Section 2.1”), which supports Sub2 loading of Mex67/MTR2 on mRNA and has been suggested to work as an alternative adaptor to Yra1 [87].

In addition to the main adaptor Yra1/AlyREF, SR proteins additionally coordinate the loading of Mex67-NXF1, providing a successful splicing checkpoint, both directly and indirectly. In yeast, Gbp2 supports recruitment of Sub2, the adaptor for Yra1, by THO during mRNP biogenesis [87], but also cooperates with Mex67 loading on mRNA via protein–protein interaction, together with Hrb1 [88]^,^ in a splicing-dependent fashion. In murine cells, the strongest direct recruitment of NXF1/Mex67 occurs via the SR proteins SRSF3 and SRSF7 [35] (see “Section 3.5”).

### 3.3. Crosstalk with Positioning of Factors on the mRNA

Even when its loading function is complete, the precise timing of THO displacement from the mature mRNP is less clear. The THO/Sub2 structure suggests that THO binding is not favoured in the closed conformation of Sub2 (when ALY loading is complete), which could mean that THO is perhaps unloaded at this step. Nevertheless, THO could remain bound to Yra1/AlyREF itself even when the latter is loaded onto the mRNP. THO could be released from the export-competent mRNP at any of these steps, but to date, the dynamics of the interaction between THO and the export-competent mRNP remain to be elucidated. As a highlight for the regulatory role of TREX-based remodelling, TREX was found to be required for transcription termination in plants [89], suggesting its key role in addressing mature RNA only for export.

### 3.4. Remodeling at the Nuclear Basket

**Figure 4:**
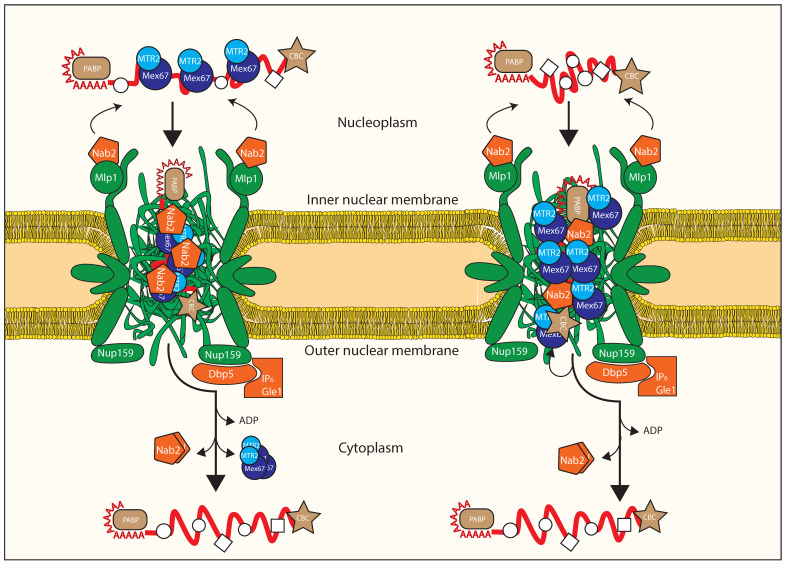
The NPC acts as a double checkpoint of mRNA export quality control. Both members of the NPC (green) and accessory proteins (orange) contribute to successful export of mature mRNP, which is characterized by capped 5′ end, polyadenylated 3′ end, and interaction with Mex67/MTR2 (in shades of blue). **Left view**: according to the classic model of mRNP export, the NPC basket member TPR/Mlp1 on the nucleoplasmic side (top) supports loading of Nab2 on the mRNP. Once the mRNP is inside the central channel, Mex67/MTR2 interacts with the FG repeats of the nucleoporins (not shown in the figure), mediating passage towards the cytoplasm. At the cytoplasmic side (bottom), anchored onto the radial-oriented Nup159, member of the Nup82 complex, Dpb5 ATPasic activity, stimulated by Gle1 and IP6, contributes to remodel the mRNP, by removing Nab2 and Mex67/MTR2. **Right view**: recent evidence suggest that Mex67/MTR2 may rather contribute to export as an integral component of the NPC. Together with loading of Nab2 by TRP/MLP (top), Mex67/MTR2 mediates interaction between the FG repeats of the nucleoporins (centre, not shown in the figure), and the mRNP, granting its passage to the cytoplasm. At the cytoplasmic side (bottom), Dpb5 removes the mRNA from Nab2 and Mex67/MTR2. It is less clear whether the latter is actively reimported or remains associated with the NPC at all times. The RNA becomes available for binding to a different set of RBPs.

As pre-mRNPs mature into export-competent mRNPs, they contact the nuclear basket of the NPC (Figure 4, top). The yeast nuclear basket is composed of Mlp1, Mlp2, Nup1, Nup2, and Nup60, which homologues are called Tpr, Nup153 and Nup50 in higher eukaryotes. Export-competent mRNPs are addressed to the NPC nuclear basket by transcription-coupled mRNA quality control, mediated by the yeast TREX-2 complex [90,91,92,93], which is stably associated with the nuclear basket in both metazoans and yeast [94,95], extensively reviewed in [96].

The role of the basket in mRNA export quality control passes by another key factor, the polyA-RNA binding protein Nab2, which is required for proper poly(A) tail length control [97] and mRNA export [98]. Nab2 is localized on the basket via its N terminal domain, that keeps it anchored to Mlp1, a basket protein (Figure 4, top) [99]. The role of the basket in quality control is proven by experiments where deletion of the yeast basket protein Mlp1 causes leakage of immature mRNAs into the cytoplasm [100]. This interplay between the mRNP and the basket is strengthened by the observation that, while on the nucleoplasmic side, Nab2 can interact directly with Mex67 [101]. Moreover, Yra1 enhances the interaction between Nab2 and Mex67, and is dispensable if Nab2 or Mex67 are overexpressed [86]. Recent evidences suggest that in yeast, Nab2 is removed from the exported transcript, together with Mex67/MTR2 [101]. Nab2 is therefore a likely adaptor for Mex67/MTR2, and strengthens the body of data that shapes Yra1 as a crucial cofactor stabilizing the export-competent mRNP in multiple ways. Interestingly, the Mlp1 human homolog, TPR, does not seem to be required for refraining aberrant mRNAs from being exported, but rather to promote efficient export of short, intronless transcripts [102].

In summary, it is increasingly clear how very many of the components of the export quality control pathway are anchored to the nuclear basket, and how many basket components have an active role in mRNA export. Together, these data strongly address the nuclear basket itself as a key hub for mRNA export quality control, as already suggested [59,103].

### 3.5. Translocation through the Central Channel of the NPC

While the protein–protein interactions occurring over the maturation process of the mRNP, and those between Mex67/MTR2 and the FG nucleoporins, have been elucidated with classical biochemistry and cell biology approaches, the basic mechanistic details of mRNP shuttling throughout the central channel of the NPC are still lacking. Experimentally, the diffraction limit for conventional light microscopy has rendered impossible any high resolution imaging of the transport process. The lack of a comprehensive mechanistic profiling of mRNA export can be at least partly ascribed to a lack of an in vitro system, which is difficult to establish due to the inherent coupling of RNA transcription and processing to its export, which leads the protein composition of mRNP to change over the course of the export process [104,105]. This coupling has also prevented to definitely associate many of the known mRNP to a clear, unique role in export, and to firmly identify factors directly involved in translocation through the NPC, as opposed to maturation RNPs that remain bound to the mRNP. How the transient interactions between the mRNP (namely via Mex67/MTR2) and the FG nups of the central channel lead to export; and how the mesh is impacted by translocation of the bulky mRNP, is technically difficult to answer.

The pioneering work on imaging RNA export involved the giant Balbiani Ring transcripts in the insect Chironomus tentans [106], which would become a model system for mRNA export due to its conveniently big size: the ca 35–40 kb gene produces in fact a 50 nm transcript. The first measurements of export kinetics revealed how the whole export process, including nuclear and cytoplasmic docking associated with mRNP quality control requires about 200 ms [107,108,109], and up to seconds for the biggest [110]. Interesting, all studies identify discrete steps, that include NPC docking, central channel translocation, and cytoplasmic release. In spite of the good accordance on the overall export time, they lack strong consensus over the relative residence time in each of these states, and lack of clarity over the rate limiting step of export. These reports have pioneered in vivo single particle imaging of mRNA export; nevertheless, the labelling strategies vary greatly, ranging from the addition of artificial stem loops in the 3′UTR region to exogenous addition of labelled non-export related RNP, without direct imaging of bound Mex67/MTR2. A new single molecule, fluorescent imaging approach has recently allowed us to restrict the field of view and allowed us to increase the resolution to ~10 nm, closer to the ~50 nm diameter of the inner central channel [111,112]. Although the mRNP labelling is achieved here with exogenous labelling as well, direct imaging of NXF1/NXT1-Tap/p15 overlapped with the mRNP observed path, consistent with the expectation of Tap/p15 as mediator of the interaction between the mRNP and the FG nucleoporins of the central channel [109]. Additionally, the low success rate observed for mRNP export (~30%) is in agreement with previous studies [107,110], in line with the role of the nuclear basket as a quality control site. A spatial probability density map generated by superposition of events in the X and Y plane revealed that mRNPs, like Tap/p15, preferentially occupy the periphery of the NPC during transport, corresponding to a radial occupancy of the central channel [109].

The current model for mRNA export, depicted on the left half of Figure 4, assumes that Mex67/Mtr2-Tap/p15 binds to a mature mRNP substrate in the nucleoplasm, and then chaperones the mRNP through the NPC channel via on-and-off interactions with FG-repeat nups within the central channel of the NPC. Two recent studies, one centred in yeast and one in human cells, address the lack of direct proof in favour of this model. Surprisingly, both agree that the bulk of the Mex67-NXF1 population is not engaged in mRNA binding, but on the contrary, they dynamically associate with members of the cytoplasmic filaments of the nuclear pore complex, confirmed by FRAP [113] and FLIM-FRET [113,114], and they do so independently from mRNA, in both studies.

Surprisingly, depletion of NPC-enriched fraction of NXF1 by overexpression of the interacting nucleoporin Nup98 resulted in export inhibition of NXF1-containing mRNPs, that were found to localize at the nuclear rim where they remained stuck. This strongly suggests that the presence of a population of NXF1 at the NPC is required for mRNA export independently of NXF1 loading on mRNA, which is further supported by an elegant rescue experiment where a Nup116-Mex67 fusion protein rescued deletion of Mex67 alone [113].

Together, these evidences back an alternative model, depicted on the right side of Figure 4, that sees Mex67-NXF1 functioning as a mobile nucleoporin: as the mature mRNP approaches the NPC, mediated by the basket-dependent platform already described, Mex67MTR2-NXF1/NXT1 interacts with the mRNP in a TREX-dependent manner once the mRNP has accessed the central channel of the NPC; the NPC-resident population of Mex67/MTR2 promotes its translocation towards the NPC exit at the cytoplasmic side. Once approaching the cytoplasmic filaments, the Nup84-Nup42 anchored Dbp5 releases the Mex67MTR2-NXF1/NXT1 from the mRNA, allowing recycling of the former and release in the cytoplasm of the latter, releasing the heterodimer from the mRNP, but likely not from the NPC.

It is unclear if, and how, the central FG mesh is impacted by the resident Mex67-NXF1 population. Little information is available about the morphology of the FG mesh per se, and there is no consensus in the field over the results obtained from structural and functional investigation of the FG-FG interaction [115,116,117,118]. Similar to what has been reported for Mex67MTR2, a population of Kap95, the bulk protein import chaperone, localizes to the nuclear rim [119,120]. In vitro studies confirmed that a Kap95 population tightly associates to a layer made by a single nucleoporin on surface plasmon resonance (SPR) experiments, which generates a collapse of the thickness of the nucleoporin layer in a way that correlates with the cohesiveness of the nucleoporin tested [121], which would help explain the discrepancy between the extremely high affinity of Kap95 for FG-repeats and the extremely fast protein import kinetics [68,122,123]. In light of this, in addition to the classic NPC barrier model, it has been suggested that karyopherins (kaps) act as integral members of the NPC [124] with a distinct group of cargo-bound, fast-translocating Kaps. Yet, unlike Mex67/MTR2-NXF1/NXT1, the affinity measurement of the Mex67^UBA^ alone for FG-domains [66] is far from the sub-nM range measured for Kap95 [68]; but most importantly, unlike Kap95, the contribution of other RBPs in the mRNP to the binding of the central FG channel has not been firmly established, nor clearly excluded.

### 3.6. Remodeling upon Exiting the Nuclear Pore Complex

Once the mRNP reaches the cytoplasmic periphery of the NPC, the directionality of transport prevents futile cycles by re-entry. Directionality is proposed to be imparted by Dbp5/DDX19, another DEAD-box ATP-dependent protein like Sub2, as a final step of nuclear mRNA export. This final step occurs by promoting release of Mex67/MTR2-Tap/p15, in an ATP-dependent manner [125,126,127,128,129,130,131,132] (Figure 4C). Removal of Mex67/MTR2 prevents re-entry of the mRNP in the nucleus, avoiding futile transport cycles, and increases Mex67/MTR2 availability to transport of a new mRNP. At the same time, in yeast, Nab2 is released as well [101] (Figure 4C). Importantly, Dbp5/DDX19 localizes stably to the cytoplasmic side of the NPC, and is part of a wider mRNA export platform that also requires scGle1/hGLE1, scNup42/hNUP42, scNup159/hNUP214, scNup116/hNUP98, and scGle2/hRAE182.

Dbp5 localization to the cytoplasmic side of the NPC occurs via interaction with the N-terminal β-propeller domain (NTD) of Nup159 [133,134,135]. On the mRNA export platform, Gle1, Nup42, and the small molecule IP6 (inositol hexakisphosphate) interact with each other, regulating Dbp5 by stimulating its ATPase activity [136,137,138,139] (Figure 4B). Dbp5 itself includes in two RecA-like domains, one on the NTD and one on the CTD, and a N-terminal extension (NTE); an alpha-helix within the NTE seems to be auto-inhibitory [140] as it occupies the region between NTD and CTD in its ADP bound state, but gets displaced when DDX19 is bound to RNA, to allow formation of a functional ATPase site. Similar self-inhibitory mechanisms have been reported in other NPC-related regulation pathways [141]. Dbp5 interaction with RNA induces ATP hydrolysis, release of Gle1, and subsequent release of Mex67/MTR2 and Nab2 as a key step. This localized cytoplasmic regulation ensures that mRNP release occurs at the NPC for newly exported transcripts, thus preventing their re-entry into the NPC. Even when Dbp5 function is impaired in mutant U2OS cells [114], mRNPs were still interacting with the NPC, confirming that Dbp5 role follows in time the transport by Mex67-NXF1 in the NPC [138].

The Nup82 complex, also located at the cytoplasmic entrance of the NPC, is another cytoplasmic NPC structure known to be predominantly involved in mRNA export [139,142,143,144,145] in a way that depends on the relative position of FG repeats in the complex [63] as the Mex67/Mtr2 heterodimer directly engages with these repeats [61,146]. Recent work highlights the role of the Nup84 subcomplex as the Dbp5 anchoring platform [147] (Figure 4B). This structural study establishes that the FG repeats of the Nup82 complex orientate towards the midplane of the central channel, where they approach the FG-repeats of Nsp1, Nup57, and Nup49 at the equator of the NPC [148,149,150].

Revisiting the classic model according to which the export disassembly platform is localized distally compared to the NPC cytoplasmic area impacts importantly our view of the disassembly process. In fact, this spatial configuration is more consistent with efficient cargo transport and remodelling, and would favour mRNP release from the central mesh of the NPC. Placing the mRNP remodelling machinery at the immediate end of the channel means that the exported mRNPs is in the right position for the Gle1/Dbp5/Nup42 re-modelling machinery to process them.

This structure also shed some light on the export machinery defects that lead to severe human diseases. Mutations in the human homolog of Gle1 are associated with lethal congenital contracture syndrome 1, lethal arthrogryposis with anterior horn cell disease [151], and amyotrophic lateral sclerosis [152]. Strikingly, the residues causing disease in human Gle1 [153] correspond to positions that this study found scGle1 tethered to either the scNup82 complex or to Nup42/Dbp5/Nup159N [147].

The dynamics of mRNA metabolism after export, and the subsequent half-life of mRNA with respect to its translation state, are relatively less understood. How the metabolism of mRNA from different genes affects the dynamics of mRNA expression is influenced by a variety of parameters, including mRNA sequence itself, cytoplasmic tail length, polyA tail-deadenylation rate, mRNP protein composition, translation rate, is a multifactorial challenge. Establishing reciprocal relationships, and the respective molecular mechanisms, between these parameters would result in a comprehensive understanding of the set of principles underlying mRNA metabolism once it has left the nucleus, ultimately contributing to establishing a structure–function connection of the RNA sequence that goes beyond the codon reading frame. The removal of the polyA tail [154,155] follows export and translation, triggering one of the two known decay pathways [156,157], which are not discussed here.

## 4. Conclusions and Future Perspectives

To date, the crew of proteins involved in mRNA export have been mostly elucidated, as well as many (but not all) of the molecular mechanisms involved. The recent developments have brought astounding progress towards molecular understanding of the structural arrangement of the machinery required for mRNA export, both belonging to the core of the mRNP or to the associated platform on the NPC, that has been reviewed.

Given the central role of mRNA export in cellular homeostasis, alterations in the chain leading to mRNA processing and transport have been linked to pathological aetiology. Diseases like Osteogenesys Imperfecta type I (OI I), myotonic dystrophy (DM), lethal congenital contracture syndrome 1 (LCCS1), lethal arthrogryposis with anterior horn cell disease (LAAHD), are some of the examples of diseases associated to different mutations of NPC-anchored nuclear export factor Gle1 and the human homolog of Mex67, NXF1. A wide variety of RBPs defects are associated with ALS [158]. Acquiring new knowledge on the basic mechanisms of mRNA export will pave the way to reinforce such protein-disease links and/or, in the future, identify new ones to further investigate.

During the lifetime of an mRNP, both conformational and compositional changes take place, either in the nucleus, during transportation through the NPC, and in the cytoplasm, prompted by the processing machinery, interaction with the central channel of the NPC, and with ribosomes, respectively. Recent, elegant studies have addressed the morphologic changes of the translating mRNP in the cytosol [7,8,159]. By contrast, information about the arrangement of the mRNP inside the nucleus is still in its infancy, and even less information is available about the journey of the mature mRNP inside the NPC. This is especially astounding considering the relatively big size of an average mRNP compared to the overall volume of the central channel—which is already occupied by the intrinsically disordered central nucleoporins. Nevertheless, these studies brought the centrality the 3D arrangement of mRNPs under the spotlight. The need for a better mechanistic understanding of mRNP morphological dynamics will require implementation of new tools that allow us to study mRNP organization, at the single-molecule level and in real time. The advancements in new bottom-up methods [160,161,162,163,164,165] will likely provide interesting new ways to look at the mechanistic details of mRNP transport in a minimal system, while it interacts with the NPC, overcoming the difficulties due to the overlapping maturation steps of export.

The finding that a pool of Mex67 is stably associated with the NPC, and that this pool provides the key shuttling function that allows mRNP translocation to the cytoplasm, is an important paradigm shift, and generates new open questions regarding the interplay between the mRNA, the NPC and Mex67. The release of Mex67 from the mRNA does not necessarily imply that the Mex67 molecules released from the mRNP leave the NPC to be recycled, as previously thought. Moreover, the implications of this finding to our understanding of the loading of Mex67/MTR2 on the mRNP (discussed in Section 3.2) are unclear, since Mex67 is able to bind RNA in the nucleus, can co-IP with mRNP export factors and is a member of mature mRNPs. The rationale behind this localization, as well as its onset; the contribution to the crowding of the FG central mesh; all these aspects remain unexplored, also considering that not all Mex67 molecules within the NPC are tending to transport of an mRNP at a given time.

Unlike protein import, mRNA export does not simply follow mRNA production and processing in time. Export steps are initiated co-transcriptionally, proceeding by addition and removal of mRNA-binding proteins. The final, export-competent mRNP is composed of a multitude of different proteins. Due to this elaborate, intertwined quality control system, the minimal required proteins within the mRNP, and nucleoporins of the FG mesh needed for the translocation through NPC have remained difficult to pinpoint clearly from accessory proteins needed for quality control and maturation. Achieving a better mechanistic understanding of multiple aspects underlying mRNP morphological dynamics will require a detailed understanding of the biophysical properties of RNPs in cells and, with it, new tools that allow us to study mRNP organization at the single-molecule level and in real time.

## Figures and Tables

**Table 1:** Crucial proteins involved in mRNA export discussed in this review.

Yeast Protein Name	Human Protein Name	Role
**mRNP Protein**		
Mex67/MTR2	NXF1/NXT1, Tap/p15	Interacts with FG nups to export mRNA
**RNA Binding Proteins—RBPs**		
Cbc1/Cbc2	Cbp20/Cbp80	Members of the Cap Binding Complex
-	SRSF1-12	SR proteins, bind mRNP during splicing
Npl3	-	SR protein, binds mRNP during splicing
Gbp2	-	SR protein, binds mRNP and contributes to loading Mex67/MTR2
Hrb1	-	SR protein, binds mRNP and contributes to loading Mex67/MTR2
**NPC—Nucleoplasmic Face**		
Sub2	UAP56	DEAD-box ATPase, member of the TREX complex, remodels mRNPs
Yra1	ALY (REF, THOC4)	Adaptor protein, member of the TREX complex
THO	THO	A pentameric complex, component of the TREX. Includes Tho2, Hpr1, Mft1, Thp2 and Tex1
Nab2	ZC3H14	Poly-A Binding protein, signals mature mRNPs for export
TREX-2	TREX-2	A multisubunit complex, anchored to the NPC basket (via Nup153 and TPR in metazoans, via Nup1 in yeast)
**NPC—Cytoplasmic Face**		
Dbp5	DDX19	DEAD-box ATPase, remodels mRNPs as they exit the NPC
Gle1	GLE1	Member of the mRNA export platform, stimulates ATPase activity of Dbp5
Nup42	NUP42	FG-nucleoporin, anchors Dbp5 and Gle1
Nup159	NUP214	FG-nucleoporin, anchors Dbp5 to the cytoplasmic side of NPC
Nup116	Nup98	FG-nucleoporin, binds Mex67/MTR2
Nup82	Nup88	Member of the Nup82 complex, mRNP remodeling platform
NSP1	Nup62	Member of the Nup82 complex, mRNP remodeling platform

## References

[1] Kopp F., Mendell J.T. (2018). Functional Classification and Experimental Dissection of Long Noncoding RNAs. Cell.

[2] Bartel D.P. (2018). Metazoan MicroRNAs. Cell.

[3] Bohnsack M.T., Sloan K.E. (2018). The mitochondrial epitranscriptome: The roles of RNA modifications in mitochondrial translation and human disease. Cell. Mol. Life Sci..

[4] Henras A.K., Plisson-Chastang C., O’Donohue M.F., Chakraborty A., Gleizes P.E. (2015). An overview of pre-ribosomal RNA processing in eukaryotes. Wiley Interdiscip. Rev. RNA.

[5] Hentze M.W., Castello A., Schwarzl T., Preiss T. (2018). A brave new world of RNA-binding proteins. Nat. Rev. Mol. Cell Biol..

[6] Youn J.Y., Dunham W.H., Hong S.J., Knight J.D., Bashkurov M., Chen G.I., Bagci H., Rathod B., MacLeod G., Eng S.W.M. (2018). High-Density Proximity Mapping Reveals the Subcellular Organization of mRNA-Associated Granules and Bodies. Mol. Cell.

[7] Adivarahan S., Livingston N., Nicholson B., Rahman S., Wu B., Rissland O.S., Zenklusen D. (2018). Spatial Organization of Single mRNPs at Different Stages of the Gene Expression Pathway. Mol. Cell.

[8] Metkar M., Ozadam H., Lajoie B.R., Imakaev M., Mirny L.A., Dekker J., Moore M.J. (2018). Higher-Order Organization Principles of Pre-translational mRNPs. Mol. Cell.

[9] Saguez C., Olesen J.R., Jensen T.H. (2005). Formation of export-competent mRNP: Escaping nuclear destruction. Curr. Opin. Cell Biol..

[10] Hershey J.W.B., Sonenberg N., Mathews M.B. (2012). Principles of translational control: An overview. Cold Spring Harb. Perspect. Biol..

[11] Gerovac M., Tampé R. (2019). Control of mRNA Translation by Versatile ATP-Driven Machines. Trends Biochem. Sci..

[12] Moore K.S., von Lindern M. (2018). RNA binding proteins and regulation of mRNA translation in erythropoiesis. Front. Physiol..

[13] Lee S.R., Pratt G.A., Martinez F.J., Yeo G.W., Lykke-Andersen J. (2015). Target Discrimination in Nonsense-Mediated mRNA Decay Requires Upf1 ATPase Activity. Mol. Cell.

[14] Baltz A.G., Munschauer M., Schwanhäusser B., Vasile A., Murakawa Y., Schueler M., Youngs N., Penfold-Brown D., Drew K., Milek M. (2012). The mRNA-Bound Proteome and Its Global Occupancy Profile on Protein-Coding Transcripts. Mol. Cell.

[15] Castello A., Fischer B., Eichelbaum K., Horos R., Beckmann B.M., Strein C., Davey N.E., Humphreys D.T., Preiss T., Steinmetz L.M. (2012). Insights into RNA Biology from an Atlas of Mammalian mRNA-Binding Proteins. Cell.

[16] Kwon S.C., Yi H., Eichelbaum K., Foehr S., Fischer B., You K.T., Castello A., Krijgsveld J., Hentze M., Kim V.N. (2013). The RNA-binding protein repertoire of embryonic stem cells. Nat. Struct. Mol. Biol..

[17] Ainger K., Avossa D., Diana A.S., Barry C., Barbarese E., Carson J.H. (1997). Transport and localization elements in myelin basic protein mRNA. J. Cell Biol..

[18] Sharma D., Zagore L.L., Brister M.M., Ye X., Crespo-Hernández C.E., Licatalosi D.D., Jankowsky E. (2021). The kinetic landscape of an RNA-binding protein in cells. Nature.

[19] Kislauskis E.H., Zhu X., Singer R.H. (1994). Sequences Responsible for Intracellular Localization of -Actin Messenger RNA Also Affect Cell Phenotype. J. Cell Biol..

[20] Blichenberg A., Schwanke B., Rehbein M., Garner C.C., Richter D., Kindler S. (1999). Identification of a cis -Acting Dendritic Targeting Element in MAP2 mRNAs Identification of a cis -Acting Dendritic Targeting Element in MAP2 mRNAs. J. Neurosci..

[21] Kiebler M.A., Desgroseillers L. (2000). Molecular Insights into mRNA Transport and Local Translation in the Mammalian Nervous System. Neuron.

[22] Raju C.S., Göritz C., Nord Y., Hermanson O., López-Iglesias C., Visa N., Castelo-Branco G., Percipalle P. (2008). In Cultured Oligodendrocytes the A/B-type hnRNP CBF-A Accompanies MBP mRNA Bound to mRNA Trafficking Sequences. Mol. Biol. Cell.

[23] Lange A., Mills R.E., Lange C.J., Stewart M., Devine S.E., Corbett A.H. (2007). Classical Nuclear Localization Signals: Definition, Function, and Interaction with Importin α. J. Biol. Chem..

[24] Eliscovich C., Buxbaum A.R., Katz Z.B., Singer R.H. (2013). mRNA on the move: The road to its biological destiny. J. Biol. Chem..

[25] Jung H., Gkogkas C.G., Sonenberg N., Holt C.E. (2014). Remote control of gene function by local translation. Cell.

[26] van Nostrand E.L., Freese P., Pratt G.A., Wang X., Wei X., Xiao R., Blue S.M., Chen J.-Y., Cody N.A.L., Dominguez D. (2020). A large-scale binding and functional map of human RNA-binding proteins. Nature.

[27] Khong A., Parker R. (2018). MRNP architecture in translating and stress conditions reveals an ordered pathway of mRNP compaction. bioRxiv.

[28] Kapeli K., Martinez F.J., Yeo G.W. (2017). Genetic mutations in RNA-binding proteins and their roles in ALS. Hum. Genet..

[29] Valkov E., Dean J.C., Jani D., Kuhlmann S.I., Stewart M. (2012). Structural basis for the assembly and disassembly of mRNA nuclear export complexes. Biochim. Biophys. Acta Gene Regul. Mech..

[30] Niño C.A., Hérissant L., Babour A., Dargemont C. (2013). MRNA nuclear export in yeast. Chem. Rev..

[31] Natalizio B.J., Wente S.R. (2013). Postage for the messenger: Designating routes for nuclear mRNA export. Trends Cell Biol..

[32] Martinez-Rucobo F.W., Kohler R., van de Waterbeemd M., Heck A.J., Hemann M., Herzog F., Stark H., Cramer P. (2015). Molecular Basis of Transcription-Coupled Pre-mRNA Capping. Mol. Cell.

[33] Cheng H., Dufu K., Lee C.-S., Hsu J.L., Dias A., Reed R. (2006). Human mRNA Export Machinery Recruited to the 5′ End of mRNA. Cell.

[34] Zahler A.M., Lane W.S., Stolk J.A., Roth M.B. (1992). SR proteins: A conserved family of pre-mRNA splicing factors. Genes Dev..

[35] Müller-McNicoll M., Botti V., Domingues A.M.D.J., Brandl H., Schwich O.D., Steiner M.C., Curk T., Poser I., Zarnack K., Neugebauer K.M. (2016). SR proteins are NXF1 adaptors that link alternative RNA processing to mRNA export. Genes Dev..

[36] Jeong S. (2017). SR proteins: Binders, regulators, and connectors of RNA. Mol. Cells.

[37] Windgassen M., Krebber H. (2003). Identification of Gbp2 as a novel poly(A)+ RNA-binding protein involved in the cytoplasmic delivery of messenger RNAs in yeast. EMBO Rep..

[38] Häcker S., Krebber P. (2004). Differential Export Requirements for Shuttling Serine/Arginine-type mRNA-binding Proteins. J. Biol. Chem..

[39] Lei E.P., Krebber H., Silver P.A. (2001). Messenger RNAs are recruited for nuclear export during transcription. Genes Dev..

[40] Krebber H., Sturm D., Windgassen M., Cajigas I., González C., Seedorf M., Bastians H. (2005). Yeast shuttling SR-proteins Npl3p, Gbp2p and Hrb1p are part of the translating mRNPs and Npl3p can function as a translational repressor. GBM Annu. Fall Meet. Berl. Potsdam..

[41] Le Hir H., Gatfield D., Izaurralde E., Moore M.J. (2001). The exon-exon junction complex provides a binding platform for factors involved in mRNA export and nonsense-mediated mRNA decay. EMBO J..

[42] Singh G., Kucukural A., Cenik C., Leszyk J.D., Shaffer S.A., Weng Z., Moore M.J. (2012). The cellular EJC interactome reveals higher-order mRNP structure and an EJC-SR protein nexus. Cell.

[43] Viphakone N., Sudbery I., Griffith L., Heath C.G., Sims D., Wilson S.A. (2019). Co-transcriptional Loading of RNA Export Factors Shapes the Human Transcriptome. Mol. Cell.

[44] Boehm V., Gehring N.H. (2016). Exon Junction Complexes: Supervising the Gene Expression Assembly Line. Trends Genet..

[45] Le Hir H., Sauliere J., Wang Z. (2016). The exon junction complex as a node of post-transcriptional networks. Nat. Rev. Mol. Cell Biol..

[46] Ares M., Grate L., Pauling M.H. (1999). A handful of intron-containing genes produces the lion’s share of yeast mRNA. RNA.

[47] Chang C.T., Hautbergue G.M., Walsh M.J., Viphakone N., van Dijk T.B., Philipsen S., Wilson S.A. (2013). Chtop is a component of the dynamic TREX mRNA export complex. EMBO J..

[48] Baejen C., Torkler P., Gressel S., Essig K., Söding J., Cramer P. (2014). Transcriptome Maps of mRNP Biogenesis Factors Define Pre-mRNA Recognition. Mol. Cell.

[49] Pandya-Jones A., Bhatt D.M., Lin C.-H., Tong A.-J., Smale S.T., Black D.L. (2013). Splicing kinetics and transcript release from the chromatin compartment limit the rate of Lipid A-induced gene expression. RNA.

[50] Mauger O., Lemoine F., Scheiffele P. (2016). Targeted Intron Retention and Excision for Rapid Gene Regulation in Response to Neuronal Activity. Neuron.

[51] Brody Y., Neufeld N., Bieberstein N., Causse S., Böhnlein E.-M., Neugebauer K.M., Darzacq X., Shav-Tal Y. (2011). The In Vivo Kinetics of RNA Polymerase II Elongation during Co-Transcriptional Splicing. PLoS Biol..

[52] Nguyen K.T., Holloway M.P., Altura R.A. (2012). The CRM1 nuclear export protein in normal development and disease. Int. J. Biochem. Mol. Biol..

[53] Carmody S.R., Wente S.R. (2009). mRNA nuclear export at a glance. J. Cell Sci..

[54] Segref A., Sharma K., Doye V., Hellwig A., Huber J., Lührmann R., Hurt E. (1997). Mex67p, a novel factor for nuclear mRNA export, binds to both poly(A)+ RNA and nuclear pores. EMBO J..

[55] Katahira J., Sträßer K., Podtelejnikov A., Mann M., Jung J.U., Hurt E. (1999). The Mex67p-mediated nuclear mRNA export pathway is conserved from yeast to human. EMBO J..

[56] Grüter P., Tabernero C., von Kobbe C., Schmitt C., Saavedra C., Bachi A., Wilm M., Felber B.K., Izaurralde E. (1998). TAP, the Human Homolog of Mex67p, Mediates CTE-Dependent RNA Export from the Nucleus. Mol. Cell.

[57] Braun I.C., Rohrbach E., Schmitt C., Izaurralde E. (1999). TAP binds to the constitutive transport element (CTE) through a novel RNA-binding motif that is sufficient to promote CTE-dependent RNA export from the nucleus. EMBO J..

[58] Aibara S., Katahira J., Valkov E., Stewart M. (2015). The principal mRNA nuclear export factor NXF1:NXT1 forms a symmetric binding platform that facilitates export of retroviral CTE-RNA. Nucleic Acids Res..

[59] Xie Y., Ren Y. (2019). Mechanisms of nuclear mRNA export: A structural perspective. Traffic.

[60] Bachi A., Braun I.C., Rodrigues J.P., Panté N., Ribbeck K., VON Kobbe C., Kutay U., Wilm M., Görlich D., Carmo-Fonseca M. (2000). The C-terminal domain of TAP interacts with the nuclear pore complex and promotes export of specific CTE-bearing RNA substrates. RNA.

[61] Sträßer K., Baßler J., Hurt E. (2000). Binding of the Mex67p/Mtr2p heterodimer to FXFG, GLFG, and FG repeat nucleoporins is essential for nuclear mRNA export. J. Cell Biol..

[62] Strawn L.A., Shen T., Wente S.R. (2001). The GLFG Regions of Nup116p and Nup100p Serve as Binding Sites for Both Kap95p and Mex67p at the Nuclear Pore Complex. J. Biol. Chem..

[63] Adams R.L., Terry L.J., Wente S.R. (2014). Nucleoporin FG domains facilitate mRNP remodeling at the cytoplasmic face of the nuclear pore complex. Genetics.

[64] Fribourg S., Braun I.C., Izaurralde E., Conti E. (2001). Structural basis for the recognition of a nucleoporin FG repeat by the NTF2-like domain of the TAP/p15 mRNA nuclear export factor. Mol. Cell.

[65] Grant R.P., Hurt E., Neuhaus D., Stewart M. (2002). Structure of the C-terminal FG-nucleoporin binding domain of Tap/NXF1. Nat. Struct. Biol..

[66] Grant R.P., Neuhaus D., Stewart M. (2003). Structural basis for the interaction between the Tap/NXF1 UBA domain and FG nucleoporins at 1 Å resolution. J. Mol. Biol..

[67] Terry L.J., Wente S.R. (2007). Nuclear mRNA export requires specific FG nucleoporins for translocation through the nuclear pore complex. J. Cell Biol..

[68] Tetenbaum-Novatt J., Hough L.E., Mironska R., McKenney A.S., Rout M.P. (2012). Nucleocytoplasmic transport: A role for nonspecific competition in karyopherin-nucleoporin interactions. Mol. Cell. Proteom..

[69] Smith C., Lari A., Derrer C.P., Ouwehand A., Rossouw A., Huisman M., Dange T., Hopman M., Joseph A., Zenklusen D. (2015). In vivo single-particle imaging of nuclear mRNA export in budding yeast demonstrates an essential role for Mex67p. J. Cell Biol..

[70] Aibara S., Valkov E., Lamers M., Stewart M. (2015). Domain organization within the nuclear export factor Mex67:Mtr2 generates an extended mRNA binding surface. Nucleic Acids Res..

[71] Beck M., Schmidt A., Malmström J., Claassen M., Ori A., Szymborska-Mell A., Herzog F., Rinner O., Ellenberg J., Aebersold R. (2011). The quantitative proteome of a human cell line. Mol. Syst. Biol..

[72] Maeshima K., Iino H., Hihara S., Funakoshi T., Watanabe A., Nishimura M., Nakatomi R., Yahata K., Imamoto F., Hashikawa T. (2010). Nuclear pore formation but not nuclear growth is governed by cyclin-dependent kinases (Cdks) during interphase. Nat. Struct. Mol. Biol..

[73] Winey M., Yarar D., Giddings T.H., Mastronarde D.N. (1997). Nuclear pore complex number and distribution throughout the Saccharomyces cerevisiae cell cycle by three-dimensional reconstruction from electron micrographs of nuclear envelopes. Mol. Biol. Cell.

[74] Sankhala R.S., Lokareddy R.K., Begum S., Pumroy R., Gillilan R.E., Cingolani G. (2017). Three-dimensional context rather than NLS amino acid sequence determines importin α subtype specificity for RCC1. Nat. Commun..

[75] Niepel M., Molloy K.R., Williams R., Farr J.C., Meinema A.C., Vecchietti N., Cristea I.M., Chait B.T., Rout M.P., Strambio-De-Castillia C. (2013). The nuclear basket proteins Mlp1p and Mlp2p are part of a dynamic interactome including Esc1p and the proteasome. Mol. Biol. Cell.

[76] Rabut G., Doye V., Ellenberg J. (2004). Mapping the dynamic organization of the nuclear pore complex inside single living cells. Nat. Cell Biol..

[77] Schuller S.K., Schuller J.M., Prabu J.R., Baumgärtner M., Bonneau F., Basquin J., Conti E. (2020). Structural insights into the nucleic acid remodeling mechanisms of the yeast THO-Sub2 complex. eLife.

[78] Batisse J., Batisse C., Budd A., Böttcher B., Hurt E. (2009). Purification of nuclear poly(A)-binding protein Nab2 reveals association with the yeast transcriptome and a messenger ribonucleoprotein core structure. J. Biol. Chem..

[79] Meinel D., Burkert-Kautzsch C., Kieser A., O’Duibhir E., Siebert M., Mayer A., Cramer P., Söding J., Holstege F.C.P., Sträßer K. (2013). Recruitment of TREX to the Transcription Machinery by Its Direct Binding to the Phospho-CTD of RNA Polymerase II. PLoS Genet..

[80] Pühringer T., Hohmann U., Fin L., Pacheco-Fiallos B., Schellhaas U., Brennecke J., Plaschka C. (2020). Structure of the human core transcription-export complex reveals a hub for multivalent interactions. eLife.

[81] Ren Y., Schmiege P., Blobel G. (2017). Structural and biochemical analyses of the DEAD-box ATPase Sub2 in association with THO or Yra1. eLife.

[82] Zhou Z., Luo M.-J., Sträßer K., Katahira J., Hurt E., Reed R. (2000). The protein Aly links pre-messenger-RNA splicing to nuclear export in metazoans. Nat. Cell Biol..

[83] Reed R., Magni K. (2001). A new view of mRNA export: Separating the wheat from the chaff. Nat. Cell Biol..

[84] Straßer K., Hurt E. (2000). Yra1p, a conserved nuclear RNA-binding protein, interacts directly with Mex67p and is required for mRNA export. EMBO J..

[85] Duncan K., Umen J.G., Guthrie C. (2000). A putative ubiquitin ligase required for efficient mRNA export differentially affects hnRNP transport. Curr. Biol..

[86] Iglesias N., Tutucci E., Gwizdek C., Vinciguerra P., Von Dach E., Corbett A.H., Dargemont C., Stutz F. (2010). Ubiquitin-mediated mRNP dynamics and surveillance prior to budding yeast mRNA export. Genes Dev..

[87] Xie Y., Clarke B.P., Kim Y.J., Ivey A.L., Hill P.S., Shi Y., Ren Y. (2021). Cryo-EM structure of the yeast TREX complex and coordination with the SR-like protein Gbp2. eLife.

[88] Hackmann A., Wu H., Schneider U.-M., Meyer K., Jung K., Krebber H. (2014). Quality control of spliced mRNAs requires the shuttling SR proteins Gbp2 and Hrb1. Nat. Commun..

[89] Khan G.A., Deforges J., Reis R.S., Hsieh Y.-F., Montpetit J., Antosz W., Santuari L., Hardtke C., Grasser K.D., Poirier Y. (2020). The transcription and export complex THO/TREX contributes to transcription termination in plants. PLoS Genet..

[90] Fischer T., Sträßer K., Racz A., Rodriguez-Navarro S., Oppizzi M., Ihrig P., Lechner J., Hurt E. (2002). The mRNA export machinery requires the novel Sac3p-Thp1p complex to dock at the nucleoplasmic entrance of the nuclear pores. EMBO J..

[91] Schneider M., Hellerschmied D., Schubert T., Amlacher S., Vinayachandran V., Reja R., Pugh B.F., Clausen T., Köhler A. (2015). The Nuclear Pore-Associated TREX-2 Complex Employs Mediator to Regulate Gene Expression. Cell.

[92] Jani D., Lutz S., Marshall N.J., Fischer T., Köhler A., Ellisdon A.M., Hurt E., Stewart M. (2009). Sus1, Cdc31, and the Sac3 CID Region Form a Conserved Interaction Platform that Promotes Nuclear Pore Association and mRNA Export. Mol. Cell.

[93] Schubert T., Köhler A. (2016). Mediator and TREX-2: Emerging links between transcription initiation and mRNA export. Nucleus.

[94] Umlauf D., Bonnet J., Waharte F., Fournier M., Stierle M., Fischer B., Brino L., Devys D., Tora L. (2013). The human TREX-2 complex is stably associated with the nuclear pore basket. J. Cell Sci..

[95] Jani D., Valkov E., Stewart M. (2014). Structural basis for binding the TREX2 complex to nuclear pores, GAL1 localisation and mRNA export. Nucleic Acids Res..

[96] Stewart M. (2019). Structure and Function of the TREX-2 Complex. Subcell. Biochem..

[97] Aibara S., Gordon J.M.B., Riesterer A.S., McLaughlin S.H., Stewart M. (2017). Structural basis for the dimerization of Nab2 generated by RNA binding provides insight into its contribution to both poly(A) tail length determination and transcript compaction in Saccharomyces cerevisiae. Nucleic Acids Res..

[98] Green D.M., Marfatia K.A., Crafton E.B., Zhang X., Cheng X., Corbett A.H. (2002). Nab2p is required for poly(A) RNA export in Saccharomyces cerevisiae and is regulated by arginine methylation via Hmt1p. J. Biol. Chem..

[99] Fasken M.B., Stewart M., Corbett A.H. (2008). Functional significance of the interaction between the mRNA-binding protein, Nab2, and the nuclear pore-associated protein, mlp1, in mRNA export. J. Biol. Chem..

[100] Galy V., Gadal O., Fromont-Racine M., Romano A., Jacquier A., Nehrbass U. (2004). Nuclear Retention of Unspliced mRNAs in Yeast Is Mediated by Perinuclear Mlp1. Cell.

[101] Adams R.L., Wente S.R. (2020). Dbp5 associates with RNA-bound Mex67 and Nab2 and its localization at the nuclear pore complex is sufficient for mRNP export and cell viability. PLoS Genet..

[102] Lee E.S., Wolf E.J., Ihn S.S.J., Smith H.W., Emili A., Palazzo A.F. (2020). TPR is required for the efficient nuclear export of mRNAs and lncRNAs from short and intron-poor genes. Nucleic Acids Res..

[103] Soheilypour M., Mofrad M.R.K. (2018). Quality control of mRNAs at the entry of the nuclear pore: Cooperation in a complex molecular system. Nucleus.

[104] Dreyfuss G., Kim V.N., Kataoka N. (2002). Messenger-RNA-binding proteins and the messages they carry. Nat. Rev. Mol. Cell Biol..

[105] Müller-Mcnicoll M., Neugebauer K.M. (2013). How cells get the message: Dynamic assembly and function of mRNA-protein complexes. Nat. Rev. Genet..

[106] Mehlin H., Daneholt B., Skoglund U. (1992). Translocation of a specific premessenger ribonucleoprotein particle through the nuclear pore studied with electron microscope tomography. Cell.

[107] Grünwald D., Singer R.H. (2010). In vivo imaging of labelled endogenous Β-actin mRNA during nucleocytoplasmic transport. Nature.

[108] Mor A., Suliman S., Ben-Yishay R., Yunger S., Brody Y., Shav-Tal Y. (2010). Dynamics of single mRNP nucleocytoplasmic transport and export through the nuclear pore in living cells. Nat. Cell Biol..

[109] Ma J., Liu Z., Michelotti N., Pitchiaya S., Veerapaneni R., Androsavich J.R., Walter N.G., Yang W. (2013). High-resolution three-dimensional mapping of mRNA export through the nuclear pore. Nat. Commun..

[110] Siebrasse J.P., Kaminski T., Kubitscheck U. (2012). Nuclear export of single native mRNA molecules observed by light sheet fluorescence microscopy. Proc. Natl. Acad. Sci. USA.

[111] Rout M.P., Blobel G. (1993). Isolation of the yeast nuclear pore complex. J. Cell Biol..

[112] Hoelz A., Debler E.W., Blobel G. (2011). The Structure of the nuclear pore complex. Annu. Rev. Biochem..

[113] Derrer C.P., Mancini R., Vallotton P., Huet S., Weis K., Dultz E. (2019). The RNA export factor Mex67 functions as a mobile nucleoporin. J. Cell Biol..

[114] Ben-Yishay R., Mor A., Shraga A., Ashkenazy-Titelman A., Kinor N., Schwed-Gross A., Jacob A., Kozer N., Kumar P., Garini Y. (2019). Imaging within single NPCs reveals NXF1’s role in mRNA export on the cytoplasmic side of the pore. J. Cell Biol..

[115] Frey S., Görlich D. (2007). A Saturated FG-Repeat Hydrogel Can Reproduce the Permeability Properties of Nuclear Pore Complexes. Cell.

[116] Li C., Goryaynov A., Yang W. (2016). The selective permeability barrier in the nuclear pore complex. Nucleus.

[117] Aramburu I.V., Lemke E.A. (2017). Floppy but not sloppy: Interaction mechanism of FG-nucleoporins and nuclear transport receptors. Semin. Cell Dev. Biol..

[118] Celetti G., Paci G., Caria J., VanDelinder V., Bachand G., Lemke E.A. (2019). The liquid state of FG-nucleoporins mimics permeability barrier properties of nuclear pore complexes. J. Cell Biol..

[119] Paradise A., Levin M.K., Korza G., Carson J.H. (2007). Significant Proportions of Nuclear Transport Proteins with Reduced Intracellular Mobilities Resolved by Fluorescence Correlation Spectroscopy. J. Mol. Biol..

[120] Tokunaga M., Imamoto N., Sakata-Sogawa K. (2008). Highly inclined thin illumination enables clear single-molecule imaging in cells. Nat. Methods.

[121] Kapinos L.E., Schoch R.L., Wagner R.S., Schleicher K.D., Lim R.Y.H. (2014). Karyopherin-centric control of nuclear pores based on molecular occupancy and kinetic analysis of multivalent binding with FG nucleoporins. Biophys. J..

[122] Ribbeck K., Görlich D. (2001). Kinetic analysis of translocation through nuclear pore complexes. EMBO J..

[123] Jovanovic-Talisman T., Zilman A. (2017). Protein Transport by the Nuclear Pore Complex: Simple Biophysics of a Complex Biomachine. Biophys. J..

[124] Lim R.Y.H., Kapinos L.E. (2015). How to operate a nuclear pore complex by kap-centric control. Nucleus.

[125] Snay-Hodge C.A., Colot H.V., Goldstein A.L., Cole C.N. (1998). Dbp5p/Rat8p is a yeast nuclear pore-associated DEAD-box protein essential for RNA export. EMBO J..

[126] Liker E., Fernandez E., Izaurralde E., Conti E. (2000). The structure of the mRNA export factor TAP reveals a cis arrangement of a non-canonical RNP domain and an LRR domain. EMBO J..

[127] Lund M.K., Guthrie C. (2005). The DEAD-box protein Dbp5p is required to dissociate Mex67p from exported mRNPs at the nuclear rim. Mol. Cell.

[128] Cole C.N., Scarcelli J.J. (2006). Unravelling mRNA export. Nat. Cell Biol..

[129] Weirich C.S., Erzberger J.P., Flick J.S., Berger J.M., Thorner J., Weis K. (2006). Activation of the DExD/H-box protein Dbp5 by the nuclear-pore protein Gle1 and its coactivator InsP6 is required for mRNA export. Nat. Cell Biol..

[130] Tran E.J., Zhou Y., Corbett A.H., Wente S.R. (2007). The DEAD-Box Protein Dbp5 Controls mRNA Export by Triggering Specific RNA:Protein Remodeling Events. Mol. Cell.

[131] Montpetit B., Thomsen N.D., Helmke K.J., Seeliger M., Berger J.M., Weis K. (2011). A conserved mechanism of DEAD-box ATPase activation by nucleoporins and InsP6 in mRNA export. Nature.

[132] Folkmann A.W., Noble K.N., Cole C.N., Wente S.R. (2011). Dbp5, Gle1-IP6 and Nup159: A working model for mRNP export. Nucleus.

[133] Schmitt C., von Kobbe C., Bachi A., Panté N., Rodrigues J.P., Boscheron C., Rigaut G., Wilm M., Séraphin B., Carmo-Fonseca M. (1999). Dbp5, a DEAD-box protein required for mRNA export, is recruited to the cytoplasmic fibrils of nuclear pore complex via a conserved interaction with CAN/Nup159p. EMBO J..

[134] Weirich C.S., Erzberger J.P., Berger J.M., Weis K. (2004). The N-terminal domain of Nup159 forms a β-propeller that functions in mRNA export by tethering the helicase Dbp5 to the nuclear pore. Mol. Cell.

[135] von Moeller H., Basquin C., Conti E. (2009). The mRNA export protein DBP5 binds RNA and the cytoplasmic nucleoporin NUP214 in a mutually exclusive manner. Nat. Struct. Mol. Biol..

[136] Alcázar-Román A.R., Tran E.J., Guo S., Wente S.R. (2006). Inositol hexakisphosphate and Gle1 activate the DEAD-box protein Dbp5 for nuclear mRNA export. Nat. Cell Biol..

[137] Dossani Z.Y., Weirich C.S., Erzberger J.P., Berger J.M., Weis K. (2009). Structure of the C-terminus of the mRNA export factor Dbp5 reveals the interaction surface for the ATPase activator Gle1. Proc. Natl. Acad. Sci. USA.

[138] Hodge C.A., Tran E., Noble K.N., Alcazar-Roman A.R., Ben-Yishay R., Scarcelli J.J., Folkmann A.W., Shav-Tal Y., Wente S.R., Cole C.N. (2011). The Dbp5 cycle at the nuclear pore complex during mRNA export I: Dbp5 mutants with defects in RNA binding and ATP hydrolysis define key steps for Nup159 and Gle1. Genes Dev..

[139] Noble K.N., Tran E., Alcazar-Roman A.R., Hodge C.A., Cole C.N., Wente S.R. (2011). The Dbp5 cycle at the nuclear pore complex during mRNA export II: Nucleotide cycling and mRNP remodeling by Dbp5 are controlled by Nup159 and Gle1. Genes Dev..

[140] Collins R., Karlberg T., Lehtiö L., Schütz P., Berg S.V.D., Dahlgren L.-G., Hammarström M., Weigelt J., Schüler H. (2009). The DEXD/H-box RNA helicase DDX19 is regulated by an α-helical switch. J. Biol. Chem..

[141] de Magistris P., Tatarek-Nossol M., Dewor M., Antonin W. (2018). A self-inhibitory interaction within Nup155 and membrane binding are required for nuclear pore complex formation. J. Cell Sci..

[142] Grandi P., Emig S., Weise C., Hucho F., Pohl T., Hurt E.C. (1995). A novel nuclear pore protein Nup82p which specifically binds to a fraction of Nsp1p. J. Cell Biol..

[143] Kraemer D.M., Strambio-de-Castillia C., Blobel G., Rout M.P. (1995). The essential yeast nucleoporin NUP159 is located on the cytoplasmic side of the nuclear pore complex and serves in karyopherin-mediated binding of transport substrate. J. Biol. Chem..

[144] Belgareh N., Snay-Hodge C., Pasteau F., Dagher S., Cole C.N., Doye V. (1998). Functional characterization of a Nup159p-containing nuclear pore subcomplex. Mol. Biol. Cell.

[145] Gaik M., Flemming D., Von Appen A., Kastritis P., Mücke N., Fischer J., Stelter P., Ori A., Bui K.H., Baßler J. (2015). Structural basis for assembly and function of the Nup82 complex in the nuclear pore scaffold. J. Cell Biol..

[146] Trahan C., Oeffinger M. (2016). Targeted cross-linking-mass spectrometry determines vicinal interactomes within heterogeneous RNP complexes. Nucleic Acids Res..

[147] Fernandez-Martinez J., Kim S.J., Shi Y., Upla P., Pellarin R., Gagnon M., Chemmama I.E., Wang J., Nudelman I., Zhang W. (2016). Structure and Function of the Nuclear Pore Complex Cytoplasmic mRNA Export Platform. Cell.

[148] Kosinski J., Mosalaganti S., von Appen A., Teimer R., DiGuilio A.L., Wan W., Bui K.H., Hagen W.J., Briggs J.A.G., Glavy J.S. (2016). Molecular architecture of the inner ring scaffold of the human nuclear pore complex. Science.

[149] Lin D.H., Stuwe T., Schilbach S., Rundlet E.J., Perriches T., Mobbs G., Fan Y., Thierbach K., Huber F.M., Collins L.N. (2016). Architecture of the symmetric core of the nuclear pore. Science.

[150] Stuwe T., Bley C.J., Thierbach K., Petrovic S., Schilbach S., Mayo D.J., Perriches T., Rundlet E.J., Jeon Y.E., Collins L.N. (2015). Architecture of the fungal nuclear pore inner ring complex. Science.

[151] Nousiainen H.O., Kestilä M., Pakkasjärvi N., Honkala H., Kuure S., Tallila J., Vuopala K., Ignatius J., Herva R., Peltonen L. (2008). Mutations in mRNA export mediator GLE1 result in a fetal motoneuron disease. Nat. Genet..

[152] Kaneb H.M., Folkmann A.W., Belzil V.V., Jao L.-E., Leblond C.S., Girard S., Daoud H., Noreau A., Rochefort D., Hince P. (2015). Deleterious mutations in the essential mRNA metabolism factor, hGle1, in amyotrophic lateral sclerosis. Hum. Mol. Genet..

[153] Folkmann A.W., Dawson T.R., Wente S.R. (2014). Insights into mRNA export-linked molecular mechanisms of human disease through a Gle1 structure-function analysis. Adv. Biol. Regul..

[154] Muhlrad D., Parker R. (1992). Mutations affecting stability and deadenylation of the yeast MFA2 transcript. Genes Dev..

[155] Shyu A.B., Belasco J.G., Greenberg M.E. (1991). Two distinct destabilizing elements in the c-fos message trigger deadenylation as a first step in rapid mRNA decay. Genes Dev..

[156] Chan L.Y., Mugler C.F., Heinrich S., Vallotton P., Weis K. (2018). Non-invasive measurement of mRNA decay reveals translation initiation as the major determinant of mRNA stability. eLife.

[157] Eisen T.J., Eichhorn S.W., Subtelny A.O., Lin K.S., McGeary S.E., Gupta S., Bartel D.P. (2020). The Dynamics of Cytoplasmic mRNA Metabolism. Mol. Cell.

[158] Zhao M., Kim J.R., Van Bruggen R., Park J. (2018). RNA-Binding Proteins in Amyotrophic Lateral Sclerosis. Mol. Cells.

[159] Fazal F.M., Han S., Parker K.R., Kaewsapsak P., Xu J., Boettiger A.N., Chang H.Y., Ting A.Y. (2019). Atlas of Subcellular RNA Localization Revealed by APEX-Seq. Cell.

[160] Caspi Y., Zbaida D., Cohen H., Elbaum M. (2008). Synthetic mimic of selective transport through the nuclear pore complex. Nano Lett..

[161] Lim R.Y.H., Huang N.-P., Köser J., Deng J., Lau K.H.A., Schwarz-Herion K., Fahrenkrog B., Aebi U. (2006). Flexible phenylalanine-glycine nucleoporins as entropic barriers to nucleocytoplasmic transport. Proc. Natl. Acad. Sci. USA.

[162] Jovanovic-Talisman T., Novatt J., McKenney A.S., Zilman A., Peters R., Rout M., Chait B.T. (2009). Artificial nanopores that mimic the transport selectivity of the nuclear pore complex. Nature.

[163] Kowalczyk S.W., Kapinos L., Blosser T.R., Magalhães T., Van Nies P., Lim R., Dekker C. (2011). Single-molecule transport across an individual biomimetic nuclear pore complex. Nat. Nanotechnol..

[164] Fisher P.D.E., Shen Q., Akpinar B., Davis L., Chung K., Baddeley D., Saric A., Melia T.J., Hoogenboom B., Lin C. (2018). A Programmable DNA Origami Platform for Organizing Intrinsically Disordered Nucleoporins within Nanopore Confinement. ACS Nano.

[165] Ketterer P., Ananth A.N., Trip D.L., Mishra A., Bertosin E., Ganji M., Van Der Torre J., Onck P., Dietz H., Dekker C. (2018). DNA origami scaffold for studying intrinsically disordered proteins of the nuclear pore complex. Nat. Commun..

